# Role of Antifungal Combinations in Difficult to Treat *Candida* Infections

**DOI:** 10.3390/jof7090731

**Published:** 2021-09-06

**Authors:** Roxana G. Vitale

**Affiliations:** 1 Consejo Nacional de Investigaciones Científicas y Tecnológicas (CONICET), Buenos Aires, Argentina; rvitale@conicet.gov.ar; 2 Unidad de Parasitología, Sector Micología, Hospital J. M. Ramos Mejía, Buenos Aires, Argentina

**Keywords:** *Candida* infections, antifungal combinations, management, amphotericin B, azoles, echinocandins

## Abstract

*Candida* infections are varied and, depending on the immune status of the patient, a life-threatening form may develop. *C. albicans* is the most prevalent species isolated, however, a significant shift towards other *Candida* species has been noted. Monotherapy is frequently indicated, but the patient’s evolution is not always favorable. Drug combinations are a suitable option in specific situations. The aim of this review is to address this problem and to discuss the role of drug combinations in difficult to treat *Candida* infections. A search for eligible studies in PubMed and Google Scholar databases was performed. An analysis of the data was carried out to define in which cases a combination therapy is the most appropriate. Combination therapy may be used for refractory candidiasis, endocarditis, meningitis, eye infections and osteomyelitis, among others. The role of the drug combination would be to increase efficacy, reduce toxicity and improve the prognosis of the patient in infections that are difficult to treat. More clinical studies and reporting of cases in which drug combinations are used are needed in order to have more data that support the use of this therapeutic strategy.

## 1. Introduction

*Candida* infections are varied, from superficial and mucocutaneous to devastating invasive disease associated with candidemia, in which any organ may be involved, such as the liver, spleen, eye, lung and skin [1,2,3,4,5,6,7,8].

Depending on the immune status of the patient, a very serious disease may develop. Patients at risk are those with immunosuppression, such as neutropenics patients, those who have undergone transplants, those with autoimmune diseases or HIV/AIDS and those undergoing the use of broad-spectrum antibiotics, central venous catheters, parenteral nutrition, surgery and medical devices (e.g., dental implants, heart valves, vascular bypass grafts, ocular lenses, artificial joints and central nervous system shunts). The latter might be substrates for biofilm formation by *Candida* species, resulting in more resistance to antifungal drugs [4,5,6,8,9,10].

In general, *C. albicans* is the most prevalent species isolated, representing 45–65% of candidemia cases. However, a significant shift towards other *Candida* species has been noted [11,12,13,14,15].

Monotherapy is frequently indicated, but the patient’s evolution is not always favorable. Therefore, what does hard to treat yeast infection mean? These are infections in which failure in the patient’s response is possible, due to different reasons that are explained below.

Therapeutic failure might be due to differences in drug bioavailability between different tissues, such as the case of less azole bioavailability in vaginal tissues due a lower pH than blood; lower bioavailability is observed in infections in the central nervous system in which adequate drug levels are not achieved by drugs that do not cross the blood–brain barrier sufficiently [16]. Another problem is biofilm formation that prevents the penetration of some antifungals [17]. Other important factors are pharmacokinetics, drug interactions and side effects. For example, due the known amphotericin B nephrotoxicity or flucytosine toxicity when used as monotherapy in the past for treating candidiasis, aspergillosis and cryptococcosis, now it has been changed and they are used mostly in combination with azoles or amphotericin B [18]. Another important factor is antifungal drug resistance. Studies have demonstrated that the overexpression of membrane transporters and ERG11 in *C. albicans* contributes to the molecular mechanisms of drug resistance in vaginal isolates, similar to the mechanisms previously seen in oral and systemic isolates [16]. In *C. glabrata*, an efflux pump is the main mechanism of resistance to fluconazole, as described in one study [19]. Antifungal drug resistance for *C. tropicalis* was related to mutations of the azole target, Erg11p, resulting in the interruption of ergosterol biosynthesis. Azole and amphotericin B resistance was observed in strains lacking functional Erg11p [20]. In *C. auris*, it was observed that older cells can accumulate and persist in hosts during chronic infections. Higher efflux in the older cells correlated with overexpression of the efflux pump encoding gene CDR, resulting in enhanced tolerance to different drugs, especially fluconazole [21,22].

In order to design better therapeutic approaches, other therapies have to be sought, such as drug combinations. One of the first combinations used was amphotericin B plus flucytosine for the treatment of cryptococcosis [23]. For mucormycosis, amphotericin B plus either azoles or caspofungin was indicated, with different outcomes [24,25]. For *Scedosporium* infections, especially in lung transplant patients with cystic fibrosis, a combination of caspofungin either with voriconazole or terbinafine is recommended and has CIII evidence (recommendation based on expert opinion and panel consensus, obtained from well-designed controlled trials without randomization) [26]. Although monotherapy is the most recommended, in cases that are more difficult to treat, either due to the isolated fungus or for any other reason such as those described, other therapeutic options should be used. Thus, the aim of this review is to address this problem and discuss the role of drug combinations in difficult to treat *Candida* infections.

## 2. Methods

A search for eligible studies published on *Candida* infections up to 30 June 2021 in PubMed and Google Scholar databases was performed, using the key words “*Candida*” AND “candidiasis”, “*Candida*” AND “candidemia”, “*Candida* fungemia”, “Candida” OR “candidiasis”, “Candidiasis treatment” AND “*C. albicans*” OR “*C. parapsilosis*” OR “*C. krusei*” OR “*C. auris*” OR “*C. glabrata*” OR “*Candida* species” AND “antifungal treatment” AND “drug combinations” AND “management” AND “combination therapy”, “*Candida*” AND “HIV”, “*Candida* endocarditis” OR “fungal endocarditis”, “*Candida* ocular infections” OR “endophtalmitis”, “*Candida* guidelines”, “mucosal candidiasis”, “vulvovaginitis” AND “candidiasis”, “oropharyngeal candidiasis”, “mucormycosis” AND “*Scedosporium*” AND “*Cryptococcus* management”.

## 3. Results

The 103 articles evaluated for eligibility were selected according to the search criteria related to the subject of the review and restricted to works published in English. Duplicates and all those articles that did not contain accurate and relevant data were excluded.

## 4. Discussion

Invasive fungal infections are increasing, resulting in significant morbidity and mortality, with *Candida* species accounting for around half of such infections [27]. *Candida* species can cause superficial infections, diaper rash candidiasis in neonates, onychomycosis, oral candidiasis, vaginitis, candidemia and systemic infections that can target any organ [1,28,29]. Candidemia is the most frequent hospital infection, accounting for up to 15% of bloodstream infections [30,31]. *Candida albicans* is the species most frequently isolated, but other species, such as *C. glabrata*, *C. tropicalis*, *C. parapsilosis*, *C. krusei*, *C.* *inconspicua*, *C. norvegensis*, *C. famata*, *C. guilliermondii*, *C. lusitaniae* and, more recently, *C. auris*, have been increasingly isolated [15,32,33]. The antifungal susceptibility profile might be different, so identification at the species level is mandatory.

In general, monotherapy is indicated [1,34]. For candidemia in neutropenic/non-neutropenic patients, echinocandins are the first choice as initial therapy. Liposomal amphotericin B is also recommended, not as a first line, with similar efficacy as micafungin. A successful outcome was observed with liposomal amphotericin B plus micafungin in a pre-term infant with *C. pulcherrima* fungemia and in an oncology patient suffering *C. guillermondii* infection [35,36]. Favorable results were reported for *C. krusei* fungemia treated with amphotericin B plus caspofungin [37]. Fluconazole can be used in patients that are not critically ill and have no previous exposure to azoles or if the *Candida* species isolated or suspected is not a resistant organism [1,34].

Drug combinations besides monotherapy are also recommended in some situations such as those depending on the type and site of infection, the patient’s condition and the species isolated. They are based on therapy guidelines, case reports or expert opinions, since clinical trials to evaluate antifungal combinations for candidiasis are scant [1,38,39]. There are several in vitro studies about the combination of antifungal drugs or antifungals with non-antifungal compounds, which are helpful to analyze synergism or antagonist action [40,41,42,43,44,45,46]. Some murine models showed a prolonged survival and reduced fungal burden for combinations such as amphotericin B plus caspofungin [47,48] or caspofungin plus posaconazole [49]. On the other hand, some trials were performed comparing different single antifungal drugs with combinations related to the patient outcome for invasive candidiasis, for which no significant difference was observed [50,51,52,53,54]. In 2003, there was a large study in which a higher rate of blood culture clearance was observed for the combination of fluconazole plus amphotericin B deoxycholate [55] but, overall, no advantage was observed with monotherapy as the first line option for treating candidemia. The advantages of combining antifungal drugs include a possible wider spectrum of drug activity, faster antifungal effect, synergy effect, lower dosing diminishes side effects and toxicity while maintaining efficacy, delay in emergence of resistant mutants and broad coverage, especially in mixed or resistant infections. There are disadvantages, such as the increment in therapy cost, possible drug reactions and antagonism, among others.

There are no accurate recommendations to combine drugs, but there are situations where treatment is more difficult, such as the case of isolating less sensitive or resistant strains, biofilm formation, infections that occur in a place of difficult drug access or when monotherapy treatment failure is observed. In these cases, combining drugs for improvement of the treatment and prognosis of the patient could be useful. There are specific recommendations related to treatment duration. In general, long therapy is indicated, no less than a month, depending on the type of infection, and it varies from patient to patient and case to case. Some special situations are mentioned below.

### 4.1. Endocarditis

Fungal endocarditis has increased in incidence, with *Candida* species being recovered in more than 90% of cases reviewed from 1995 to 2002 [56], and with a high mortality rate [57]. Risk factors are patients with severe immunodeficiency, patients who underwent valvular surgery, patients with persistent candidemia or intravenous drug users. For native and prosthetic valve endocarditis, amphotericin B lipid formulation 3–5 mg/kg daily, caspofungin 70–50 mg/d with or without flucytosine 25 mg/kg 4 times daily or high-dose echinocandins (caspofungin 150 mg daily, micafungin 150 mg daily or anidulafungin 200 mg daily) are recommended for initial therapy plus valve replacement [1,34,58,59,60]. From a meta-analysis of medical versus surgical therapy for *Candida* endocarditis, it was observed that drug combinations such as amphotericin B plus flucytosine might provide an advantage over monotherapy for those patients in whom surgery is not an option [61]. In patients with pacemakers, removal of the device appears mandatory [62]. In a case of mural endocarditis, which is the inflammation and disruption of the non-valvular endocardial surface of the cardiac chambers, a successful treatment combining caspofungin and voriconazole in a non-operated patient was reported [63]. In a report of *C. glabrata* prosthetic mitral valve endocarditis, the initial treatment was fluconazole monotherapy. After 8 days, the cultures remained positive. Caspofungin was added and the clearance of the yeast was observed after 41 days of i.v. fluconazole and 34 days of caspofungin. Thus, a successful outcome with that combination without surgery was observed in a patient with kidney disease [64]. The combination of liposomal amphotericin B plus caspofungin was prescribed in an elderly patient with native valve endocarditis due to *C. glabrata*, resulting in a successful outcome, without surgery after nine months of follow-up [65]. However, in one study of fifteen patients with endocarditis, three were unsuccessfully treated with caspofungin combined either with liposomal amphotericin B or voriconazole or itraconazole [66]. It has been hypothesized that in some cases the antifungal drugs might have no action due the late appearance of symptoms and vegetations that are visible by echocardiograph after several months of the initial candidemia might not be detected due to the low sensitivity of blood cultures if there is postoperative transitory candidemia (Table 1).

Overall, diagnosis is very challenging as most of the time, blood cultures are negative or take a long time to yield growth. Thus, fungal endocarditis demands a high suspicion, surveillance of risk factors, fast diagnosis by echocardiography, multiple blood cultures and surgery whenever feasible. The high mortality rate and complexity of these patients results in a lack of prospective randomized clinical trials that are critical to ascertain the best course of therapy. The first choice is amphotericin B with or without flucytosine and surgery. Despite the fact that amphotericin B is recommended, reduced activity against *Candida* biofilm and failure to penetrate well into fibrin clots and vegetations have been observed [61,79]. Contrarily, caspofungin has been shown to have excellent activity in biofilms, so it is a good therapeutic option [61,80,81]. Fluconazole, 400–800 mg/d, is recommended as long-term suppressive therapy. However, caution should be taken depending on the species isolated, such as *C. glabrata* or *C. krusei*, for which other antifungals will be better options. Moreover, as was discussed, in selected patients in whom surgical therapy is not possible, combination therapy can optimize the chance for treatment success.

### 4.2. Central Nervous System

*Candida* infections in the nervous system in adults can occur, especially due to a disseminated candidiasis, as a complication of a neurosurgical procedure or as a chronic infection. Meningitis and brain abscess might appear. The combination of liposomal amphotericin B 3–5 mg/kg daily or fluconazole plus flucytosine 25 mg/kg 4 times daily is recommended [1,34]. Excellent flucytosine levels are achieved in CSF. Moreover, this combination showed in vitro synergism and it is known and widely used for cryptococcal meningitis with good results [23]. Fluconazole has excellent CSF levels, but is not recommended as initial therapy for *Candida* meningitis, since only CIII evidence was reported for high doses. Thus, this therapy is used in patients for whom amphotericin B is contraindicated [1]. For the rare cases of *C. glabrata* or *C. krusei* meningitis, voriconazole seems to be appropriate therapy after initial treatment with amphotericin B and flucytosine [1]. Cases of *C. albicans* meningitis in infants were reported with a successful outcome when treated with amphotericin B plus flucytosine [82,83,84]. In a report of HIV patients and drug abusers with a CD4 count of 100 cells/mm^3^, *Candida* meningitis was diagnosed in six patients, and successfully treated with a combination of amphotericin B plus flucytosine [67]. It is worth mentioning that one should be aware of the suspicion of *Candida*, as the CSF in this case shows mild pleocytosis and hypoglycorrhachia, parameters that are indistinguishable from those seen in tuberculous or cryptococcal meningitis [67]. In a report of *Candida* meningitis diagnosed in 17 patients, including ten children and seven adults, the treatment was amphotericin B deoxycholate or fluconazole alone, or a combination of intravenous amphotericin B with intrathecal amphotericin B, flucytosine or fluconazole. All indwelling central nervous system devices were externalized or removed. Four deaths were observed in the adult population. Three of them had received less than 48 h of antifungal agents and the other died despite fluconazole therapy for 31 days [68] (Table 1).

Overall, for CNS *Candida* involvement, no strong recommendation can be given, due to a lack of data. High suspicion, risk factors and laboratory findings are important. The most recommended treatment is liposomal amphotericin B 3–5 mg/kg daily combined with flucytosine 25 mg/kg 4 times daily for adults and, for infants, deoxycholate amphotericin B, 1 mg/kg/d or liposomal amphotericin B, 5 mg/kg/daily plus flucytosine, 25 mg/kg 4 times daily. Whenever feasible, the devices should be removed.

### 4.3. Ocular Candidiasis

There are two named forms: chorioretinitis, which is the inflammation of the choroid and the retina, and endophthalmitis, which is the inflammation of the vitreous body. Ocular candidiasis may cause pain, disturbed vision or poor visual outcomes since the diagnosis is often made in late stages. For endophthalmitis, the majority of published cases report the use of intravenous and/or intravitreal deoxycholate amphotericin B with or without oral flucytosine as initial therapy, as well as monotherapy with fluconazole, lipid amphotericin B or voriconazole [1,34]. In one study with six patients with endophthalmitis, *C. albicans* and *C. glabrata* were isolated in blood culture. Treatment began with fluconazole, but was switched to a combination of voriconazole and caspofungin, administered orally, intravitreally or i.v. as appropriate. All patients resolved the ocular infection and survived, except one [69]. However, the rationale for using combinations is unclear and it is not known whether any of these antifungals alone would also be effective. Intravitreal injection of amphotericin B deoxycholate 5–10 µg dissolved in 0.1 mL of sterile water is the standard approach, combined with systemic antifungals and surgery [70,71]. Successful use of fluconazole concomitant with systemic amphotericin B deoxycholate has been reported. In advanced disease, surgery with intraocular amphotericin B deoxycholate and systemic fluconazole has been applied successfully [70,71]. More recently, intravitreal voriconazole has been evaluated, and in animal models, doses of 25 mg/L vitreously, that is, 100 µg absolute in an adult human eye, were found to be safe [85]. Published cases were frequently treated with combined approaches, and the efficacy of voriconazole monotherapy has not yet been defined [86]. A combination of topical antifungals, amphotericin B with natamycin and voriconazole, followed by amphotericin B was reported in cases of keratitis [72] (Table 1).

Overall, in addition to monotherapy with azoles or amphotericin B, many cases have been successfully treated with combinations of drugs (systemically or by intravitreal injection) over the long term. Moreover, surveillance of patients with candidemia is recommended and fundoscopy has to be performed. Risk factors should also be considered, such as the use of topical corticosteroids, substance abuse, contact lens use, preexisting ocular surface diseases or ocular trauma.

### 4.4. Bone and Joint Candidiasis

This group includes osteomyelitis, arthritis and prosthetic joint infection. No randomized clinical trials are available, and the best therapeutic approach is poorly known. Treatment with azole monotherapy, amphotericin B or caspofungin is recommended [34]. However, in some cases of *Candida* osteomyelitis, most experience has been gathered with amphotericin B formulations, combined with flucytosine, or flucytosine plus fluconazole/voriconazole/caspofungin, generally followed by fluconazole in the long term and surgical debridement when necessary [73,74]. Successful treatment with the combination of caspofungin, 70 mg then 50 mg/24 h and flucytosine, 2.5 g/12 h was observed in a patient with knee arthritis due to *C. glabrata* [75]. Another report describes the favorable outcome of a patient with *C. krusei* lumbar spine infection treated with a combination of caspofungin and posaconazole [76] (Table 1).

Overall, more data are available on monotherapies, but on a case-by-case basis, a combination of antifungals may be an adequate option. It is important to be aware of risk factors for osteomyelitis such as the presence of a central venous catheter, antibiotic use, osteoarticular pain, previous history of *Candida* infection, immunosuppression, candidemia or IV drug use. In arthritis cases, open drainage and prosthesis removal is advisable.

### 4.5. Mucosal Candidiasis

In general, mucosal candidiasis is treated with a single drug, but in some refractory cases, drug combinations might be more effective. For oropharyngeal candidiasis, monotherapy is recommended, especially with fluconazole, when non-resistant strains are involved [1,78]. In a small trial with twenty patients with *Candida* esophagitis, the combination of amphotericin B plus flucytosine showed no advantage compared with fluconazole alone [87]. However, some reports suggest the use of antifungal combinations, especially in refractory cases. A combination of fluconazole and terbinafine was shown to clear oropharyngeal candidiasis in a refractory patient with AIDS, first treated with fluconazole [77,88].

Vulvovaginitis is a vaginal infection caused by *Candida* spp. that affects 70 to 75% of women at least once during their lives, and recurrent vulvovaginal candidiasis is often observed. Treatment focuses on the use of topical or oral fluconazole [1]. However, in complicated vulvovaginitis (such as the case of recurrent vulvovaginitis), biofilm production or cases caused by non-albicans species, other alternatives are recommended. They includes topical 17% flucytosine cream alone or in combination with 3% amphotericin B cream administered daily for 14 days or nystatin vaginal cream combined with flucytosine [1,78]. A topical combination of miconazol plus domiphen bromide was demonstrated to be effective in a vulvovaginitis animal model in rats [89]. Symptoms of bacterial vaginosis and vulvovaginal candidiasis are similar, thus a rapid, point-of-care test to distinguish between the two presentations would be of great value in treatment decision making (Table 1).

### 4.6. Candiduria

For pyelonephritis, fluconazole at a dosage of 200–400 mg/3–6 mg/kg daily for 2 weeks is recommended. For patients with fluconazole-resistant *Candida* strains, especially *C. glabrata*, alternatives include amphotericin B 0.5–0.7 mg/kg daily with or without flucytosine, 25 mg/kg 4 times daily [1]. Fungus balls can occur anywhere in the urinary system. Surgical intervention is strongly recommended plus fluconazole 200–400 mg/d. Amphotericin B 0.5–0.7 mg/kg daily with or without flucytosine 25 mg/kg 4 times daily is an alternative. Treatment duration should be until symptoms have resolved and urine cultures are negative [1] (Table 1).

### 4.7. Infections Due to Less Susceptible Candida

There are some species that are reported to be resistant or less susceptible to antifungal drugs.

*C. auris* (related to *C. haemulonii*) was reported to be resistant or less susceptible to fluconazole, amphotericin B and echinocandins and resistance to two and three antifungal classes was also observed [90,91]. Echinocandins are the first line therapy for *C. auris* infection. In cases of unresponsiveness to echinocandins, liposomal amphotericin B (as single or combination therapy with an echinocandin) should be prescribed [39]. In vitro synergy of micafungin plus amphotericin B, sulfamethoxazole or lopinavir plus azoles was observed against *C. auris* as well echinocandins plus azoles with no antagonism effect [92,93,94,95]. In patients with poor outcome initially treated with an echinocandin, a second antifungal agent, such as liposomal amphotericin B or isavuconazole, was recommended to be added [96]. A combination of amphotericin B and micafungin (either with voriconazole or amphotericin B) was shown to reduce mortality in neonatal infections [97]. In 2018, a candidemia outbreak in a Spanish hospital was reported, having a second peak in 2020. *C. auris* has displaced *C. glabrata* and *C. parapsilosis*. From 44 patients, half were treated with echinocandins only and half with a combination of echinocandins either with amphotericin B or isavuconazole [98].

*C. glabrata* and related species (*C. bracharensis*, *C. nivariensis*), *C. guilliermondii* and *C. parapsilosis* and related species (*C. metapsilosis*, *C. orthopsilosis*) might have increased rates of resistance to echinocandins due the “hot spot” FKS mutations and are often associated with decreased susceptibility to azoles, particularly to fluconazole [99,100,101]. *C. lusitaniae*, despite usually being susceptible to echinocandins, was reported to have acquired resistance during persistent candidemia in an immunocompromised child [102]. In vitro testing for echinocandin resistance and drug combinations for *C. glabrata*, *C. parapsilosis* and *C. auris* isolates could be helpful to observe if synergy or antagonism effects exist [100,103]. Combined therapy might reduce events when compared to either drug type used as monotherapy. The different antifungal drugs exhibited different mechanisms of action and when they are combined they might improve in their access to the target, even when used simultaneously or sequentially. Combined therapy is important for developing optimal management strategies, avoiding drug interactions and toxicity and improving the bioavailability. Thus, for infections where resistant or less sensitive species such as those mentioned above are isolated, an indication of combined drugs may be considered, especially if no improvement is observed with an initial monotherapy treatment.

## 5. Conclusions

Data from clinical trials regarding the use of antifungal combinations for *Candida* infections are scant. More data are available from in vitro and some animal model studies. There is a limited recommendation from guidelines in special situations such *Candida* meningitis or endocarditis. The classical combination is amphotericin B plus flucytosine, although it is no longer available worldwide. Other recommendations are from reported cases or expert opinions. Combination therapy can be rationally given due the advantages such as the diminution of toxicity or fewer drug interactions. Patients should undergo close follow-up to detect therapeutic failure and microbiological culture for identification at the species level. In addition, biofilm formation by *Candida* species is an issue since drug sequestration in the matrix reduces the drug efficacy and also provides an environment of lower exposure that may facilitate selection for acquired resistance.

In conclusion, the role of the drug combination would be to increase efficacy, reduce toxicity and improve the prognosis of the patient in infections that are difficult to treat, either because the site of infection is difficult to reach, because a resistant or less sensitive strain is isolated or because the patient suffers from a refractory infection with poor clinical outcome. Combined therapy is important for developing optimal management strategies. More clinical studies and reporting of cases in which drug combinations are used are needed in order to have more data to support the use of this therapeutic option.

## Figures and Tables

**Table 1:** Main antifungal combinations used for difficult to treat *Candida* infections.

Disease	Therapy	References (from Guidelines)	References (from Case Reports)
Native prosthetic valve endocarditis	LAMB 3–5 mg/kg/d CAS 70–50 mg/d +/− 5FC 25 mg/kg/4 times/d CAS/MCF 150 mg/AND 200 mg plus surgery	[1,34]	
Mural endocarditis	CAS 70–50 mg/d + VCZ 6mg/kg/d/12 h		[63]
Prosthetic mitral valve endocarditis	FCZ 400 mg/iv + CAS 70–50 mg/d		[64]
Native valve endocarditis	AMBd 4 g total dose + CAS 100–50 mg/d		[65]
Central nervous system	LAMB 3–5 mg/kg/d or FCZ + 5FC 25 mg/kg/4 times/d	[1,34]	
*Candida* meningitis	AMBd/LAMB + 5FC AMBd 0.5–1 mg/kg/d i.v./intrathecal 0.025–0.4 mg + 5FC 30–120 mg/kg/d or FCZ 3–8 mg/kg/d		[67,68]
Endophthalmitis	AMBd i.v. or intravitreal +/− 5FC	[1,34]	
Endophthalmitis	VCZ + CAS		[69]
Endophthalmitis	AMBd intravitreal injection 5–10 µg + systemic ATF		[70,71]
Keratitis	AMB + NAT + VCZ		[72]
Osteomyelitis	AMBd/LAMB + 5FC or 5FC + FCZ/VCZ/CAS		[73,74]
Knee arthritis	CAS 70–50 mg/d + 5FC 2.5 g/12 h		[75]
Lumbar spine infection	CAS + PCZ		[76]
Oropharyngeal candidiasis	FCZ + TBF		[77]
Vulvovaginitis	Topical 17% 5FC cream + 3% AMB cream/d NYS vaginal cream + 5FC	[1]	[78]
Candiduria (fungus ball)	AMB + 5FC	[1]	

AMBd: deoxicholate amphotericin B. LAMB: liposomal amphotericin B. 5FC: flucytosine. CAS: caspofungin. MCF: micafungin. AND: anidulafungin. FCZ: fluconazole. NYS: nystatin. VCZ: voriconazole. PCZ: posaconazole. TBF: terbinafine. NAT: natamycin.

## Data Availability

All data are publicly available.
